# Progress in Surgery

**Published:** 1899-01-14

**Authors:** 


### PROGRESS IN SURGERY.

### GENITO-URINARY SURGERY.

(*Continued from page* 249.)

**Septic Infection** of the urinary tract is a subject to which attention has been devoted of late. Dr. David Newman opened a discussion thereon, saying that the infection might be by the ureters or from the blood or lymph.[8] It was pointed out that two groups of organisms may be present—urea-decomposing cocci, and the bacillus coli. The surgeons present differed as to the relative importance to be attached to each. Mr. Mansell Moullin regards catheter or urinary fever as in all cases due to sepsis.[9] A rigor is not at all a necessary accompaniment. Many cases are insidious, the patient gradually developing symptoms of septicæmia with partial suppression, followed by drowsiness, coma, and death. The earliest and constant sign is cystitis with a cloud of pus and bacteria (streptococcus pyogenes and b. coli). The micro-organisms are always present in cystitis, and are always absent from healthy urine in the bladder. Micro-organisms are introduced into the urine as it passes out over the urethra, or by the passage of an instrument as it passes in. With a healthy bladder, which expels all its urine, little or no harm is usually done by introducing organisms, but the case is very different when the bladder is diseased. Alkalinity of urine is not a true test of cystitis; the worst forms may occur with an acid urine, usually a virulent form of b. coli being present. These facts are embodied in the author's book.[10] He advocates early cystotomy and drainage, preferably by the suprapubic route.

**Contracted Bladder** with chronic cystitis has been treated by Dr. H. Young by means of hydraulic pressure of bland fluids at a height of from 4 to 7 feet.[11] In his hands the method has proved successful, and he seems to have had no bad results, although there is a theoretical risk of renal infection.

**Tumours.**—Dr. Newman (Glasgow) describes four cases in which the local treatment of bladder tumours has been carried out by Leiter's and Howard Kelly's cystoscopes. One interesting case of angeioma of the bladder he cured by electrolysis through Howard Kelly's speculum.

**Hernia of the Bladder**, according to Brunner, occurs in as many as 1 per cent. of cases for radical cure of hernia. The gravity of the complication depends chiefly upon whether the bladder is wounded or not; hence care should be taken to avoid opening it, and if opened accidentally it should be very carefully sutured.[12]

**The Vesiculæ Seminales** have been brought within the scope of surgery by a free transverse perineal incision, so that it is now possible to remove them, together with the vasa deferentia, for tuberculous disease.[13]

**Ectopia Vesicæ.**—Reginald Harrison's case, in which he excised one kidney and transplanted the ureter of the other kidney has proved fatal from septic nephritis. Fowler's case of transplantation of the ureters into the rectum was well two years after operation.

**Prostate.**—In prostatic hypertrophy the length of the urethra averages more than eight inches, and the increase may be an important element in diagnosis.[14] Bottini's operation has been advocated by W. Meyer and by Dr. H. Morton.[15] The latter emphasises the danger of all cutting operations in old men, whereas Bottini's operation simply divides the obstructing bar, and by the heat of the cautery seals against infection. It is particularly useful when there is an obstructing bar rather than an obstructing lobe. The instrument is like a lithotrite, the male blade of which is heated by a powerful electric current, and, by a small screw, is made to cut its way through the prostate. The mortality of the operation is comparatively low. It is unsuited to the adenomatous forms of enlargement for which castration or vasectomy are more suitable.

When stone complicates enlargement of the prostate litholapaxy should be first considered, and if this be deemed inadvisable suprapubic lithotomy and possibly prostactectomy should be performed.[16]

**Urethral Stricture.**—The one great difficulty associated with the excellent operation of Wheelhouse is the difficulty of finding the proximal end of the urethra in many cases. Rather than abandon the attempt, it is better to cut down on the bladder above the pubes and pass a catheter into the urethra from within, which at once gets over the difficulty. In difficult cases with perineal fistulæ, &c., this may be done first, the point of the catheter or bougie acting as a guide to be cut down upon where it is arrested behind the stricture. Then the urethra is opened in front of the stricture by the usual Wheelhouse method; and lastly, the stricture itself is divided from the front.[17] By tying a cord to the catheter and bringing it out of the wound provision is made for changing the catheter with certainty.

**Urethritis.** — Dr. J. Cohn having proved the sterility of the normal posterior urethra, proceeded to investigate the bacteriology of chronic posterior urethritis. In none of the 12 cases examined did he find the gonococcus. In 11 of the cases he found the staphylococcus albus, and in four of these they were associated with other bacteria. Probably, most surgeons will agree with Cathcart that warts are not due to the gonococcus, but are infectious tumours acquired by contact.[18]

**Syphilis.**—Van Niessen (Wiesbaden) has contributed some important views as to syphilis. He states that the contagium of syphilis is in every case and in every stage demonstrable by microscope and by cultivation, and that with the therapeutic means known up to this time syphilis is absolutely incurable.[19] Professor

Adami gives expression to what many surgeons feel, that there is no valid distinction to be drawn between secondary and tertiary syphilis.[20] It seems that the prevailing opinion is that syphilis can only be rendered latent, not cured, and that in high degrees of virulence it is infectious in the tertiary stage.

Attention is drawn to the occasional occurrence of osteo-periostitis before the initial roseola occurs, the lesions being very diffuse.[21]

[8] Lancet, Aug. 6, 1898, p. 356. [9] Lancet, Sept. 10, 1898, p. 682. [10] Inflammation of the Bladder, Moullin, 1898. [11] Lancet, July 16, 1898, p. 160. [12] Epit. Brit. Med. Jour., July 30, 1898. [13] Practitioner, July, 1898. [14] Keyes, Amer. Jour. Med. Sci., Aug., 1898. [15] New York Med. Rec., Mar. and Sept., 1898. [16] Keyes, Ann. of Surg., May, 1898. [17] Birmingham Med. Rev., Aug., 1898. [18] Epit. Brit. Med. Jour., Sept. 3, 1898. [19] Internat. Med. Mag., July, 1898. [20] New York Med. Jour., July 16, 1898, p. 105. [21] Etienne (Jour. de Med.), July 25, 1898.

